# Is the Inherent Potential of Maize Roots Efficient for Soil Phosphorus Acquisition?

**DOI:** 10.1371/journal.pone.0090287

**Published:** 2014-03-03

**Authors:** Yan Deng, Keru Chen, Wan Teng, Ai Zhan, Yiping Tong, Gu Feng, Zhenling Cui, Fusuo Zhang, Xinping Chen

**Affiliations:** 1 Center for Resources, Environment and Food Security, China Agricultural University, Beijing, China; 2 State Key Laboratory for Plant Cell and Chromosome Engineering, Institute of Genetics and Developmental Biology, Chinese Academy of Sciences, Beijing, China; 3 State Key Laboratory of Soil Erosion and Dryland Farming on the Loess Plateau, Institute of Soil and Water Conservation, Chinese Academy of Sciences and Ministry of Water Resource, Yangling, China; University of Nottingham, United Kingdom

## Abstract

Sustainable agriculture requires improved phosphorus (P) management to reduce the overreliance on P fertilization. Despite intensive research of root adaptive mechanisms for improving P acquisition, the inherent potential of roots for efficient P acquisition remains unfulfilled, especially in intensive agriculture, while current P management generally focuses on agronomic and environmental concerns. Here, we investigated how levels of soil P affect the inherent potential of maize (*Zea mays* L.) roots to obtain P from soil. Responses of root morphology, arbuscular mycorrhizal colonization, and phosphate transporters were characterized and related to agronomic traits in pot and field experiments with soil P supply from deficiency to excess. Critical soil Olsen-P level for maize growth approximated 3.2 mg kg^−1^, and the threshold indicating a significant environmental risk was about 15 mg kg^−1^, which represented the lower and upper levels of soil P recommended in current P management. However, most root adaptations involved with P acquisition were triggered when soil Olsen-P was below 10 mg kg^−1^, indicating a threshold for maximum root inherent potential. Therefore, to maintain efficient inherent potential of roots for P acquisition, we suggest that the target upper level of soil P in intensive agriculture should be reduced from the environmental risk threshold to the point maximizing the inherent potential of roots.

## Introduction

Modern high-intensity agriculture strongly relies on phosphorus (P) fertilization [1], [2], but sustainable agriculture requires improved P management. On one hand, there is increasing concern about P scarcity because the world’s main source of P fertilizers, phosphate rock, is a limited and non-renewable resource [3], [4], [5], [6]. On the other hand, P fertilization can harm the environment by contributing to the eutrophication of water bodies [7], [8]. Inappropriate P fertilization accelerates the soil P imbalance in croplands worldwide [9].

P is readily fixed in most soils and has low availability to plants [10]. To enhance P acquisition, plants and their root-associated microbes have evolved a series of strategies that include modified root growth and functioning. Common strategies about root growth are increased root/shoot ratio [11], [12], modified root architecture [13], [14], decreased root diameter [15], enhanced specific root length (root length per unit root mass) [16], higher root hair length and/or density [17], [18], and production of aerenchyma [13], [19]. These morphological adaptations can greatly enhance the volume of soil root will exploit, and/or benefit exploitation of P-rich patches [20]. Also associations with arbuscular mycorrhizal (AM) fungi greatly extend the soil exploration space beyond the roots for many higher plant species [21]. Besides increasing soil volume exploited, roots and associated microbes can increase P availability from touched inorganic and organic sources by enhancing synthesis and exudation of organic acids and phosphatases [15], [22]. Increased P-uptake capacity by enhancing expression of high-affinity phosphate (Pi) transporters is another typical response of root functioning to facilitate P acquisition [23].

Current P management in intensive agriculture focuses on agronomic and environmental concerns, aiming to maintain soil P level between critical values that maximize crop yield but minimize P loss [24]. Most research, which explores the inherent potential of roots for efficient P acquisition, has focused on the adaptive mechanisms of P-efficient plants under P deficiency in natural ecosystems or low-yielding agricultural systems [13], [25], [26], [27]. In intensive agriculture, where soil P supply is increased by fertilization, the inherent potential of roots for efficient P acquisition is unfulfilled. In addition, interactions of P between soil and plants have often been studied in controlled and short term experiments not representative of field cropping systems.

Here, we hypothesized that the inherent potential of roots can be manipulated by managing soil P level to achieve a soil P supply that maximizes root uptake of P, optimizes crop yield and minimizes P loss. In pot and field experiments, we investigated root morphology, AM colonization, and expression of Pi transporter genes in maize (*Zea mays* L.) with different levels of P supply. The responses of these root traits were related to agronomic traits, and the optimum soil P supply was estimated.

## Materials and Methods

### Pot experiment

A pot experiment was carried out in the glasshouse at China Agricultural University from April to June, 2011. Maize (NE15, a test-cross variety) plants were planted in a calcareous silt loam soil which was collected from the same experimental site that was used for the field study (see next section). The initial soil properties were: pH 8.35 (1 : 5 soil : water ratio), organic matter 7.09 g kg^−1^, total N 0.51 g kg^−1^, Olsen-P 1.19 mg kg^−1^, and exchangeable K 90 mg kg^−1^. Seven P application rates (0, 12.5, 15, 50, 75, 100 and 300 mg P kg^−1^ soil) were used with P added as calcium superphosphate. In addition, N and K (as urea and potassium sulfate, respectively) were each applied at 150 mg kg^−1^ soil. Soil was air-dried and ground to pass through a 2-mm sieve. The added nutrients were mixed well with 8 kg of soil and filled into 4.5-L pots, with six replicate pots per treatment. Maize seeds were surface sterilized (30 min in a 10% H_2_O_2_ solution), rinsed, imbibed (8 h in a saturated CaSO_4_ solution), and germinated in a dark and humid environment for 24 h. Two germinated seeds were planted per pot, and the seedlings were thinned to one per pot at the 3-leaf stage.

Plants were harvested 56 days after planting (DAP) at the 8-leaf stage. In each treatment, three pots were randomly chosen for shoot, root and soil sampling. Shoots were removed, oven-dried at 60°C for three days, weighed, and ground for nutrient analysis. All visible roots in each pot were carefully picked out by hand and stored in an ice box before transferring to the laboratory, after which soil samples were taken. In the laboratory root samples were carefully cleaned with tap water and frozen at −20°C before measurement of root morphology and AM colonization. Soil samples were air-dried and ground to pass through a 1-mm sieve for analysis of soil Olsen-P and CaCl_2_-P. The remaining three pots in each treatment (except pots treated with 75 mg P kg^−1^ soil) were sampled for analysis of expression of Pi transporters in roots. Plant roots were gently taken out and immediately washed and then stored in liquid nitrogen for later RNA extraction.

### Field experiment

The field experiment was conducted at the Shangzhuang Experimental Station of China Agricultural University (40°8′27″N, 116°10′39″E) in Beijing. This site is located in the northern North China Plain and has a typical semi-humid monsoon climate of the warm temperate zone. The annual average temperature ranges from 11 to 13°C, and annual rainfall ranges from 480 to 580 mm with precipitation mainly occurring from June to August. Annual mean sunshine is 2750 h, and 180–200 days are frost-free. Similar to the pot experiment, the soil in the field experiment was silt loam and with these properties at 0–30 cm depth: pH 8.00 (1 : 5 soil : water ratio), organic matter 8.02 g kg^−1^, total N 0.37 g kg^−1^, Olsen-P 1.82 mg kg^−1^, and exchangeable K 82 mg kg^−1^.

The treatments consisted of eight P application rates (0, 12.5, 25, 50, 75, 100, 150 and 300 kg P ha^−1^) with four replicate plots per rate in a randomized complete block design. Each plot had an area of 17.5 m^2^ (5 m×3.5 m). Before sowing, the entire quantity of P ( as calcium superphosphate), 75 kg N ha^−1^ (as urea), and 62 kg K ha^−1^ (as potassium sulfate) were broadcast and mixed into top 20 cm of soil by disking. At 6-leaf and 13-leaf stages, an additional 150 kg N ha^−1^ as urea was top-dressed to each plot, with 75 kg N ha^−1^ each time. The same maize variety as that in pot experiment was used and seeds were planted at 67,500 plants ha^−1^ on 6 June, 2011.

At the flowering stage (57 DAP), three plants per plot were harvested from all plots and separated into shoots and roots. The shoot samples were used for determination of shoot dry weight and P content. Root samples were washed for assessment of AM colonization. Root samples for analysis of morphological traits were collected using the monolith method (see below) in all replicates of the following six P treatments: 0, 12.5, 25, 50, 100 and 300 kg P ha^−1^. With the shoot at the center, each monolith measured 40 cm perpendicular to the rows (row spacing was 60 cm)×20 cm parallel with the rows (plant spacing was 25 cm)×30 cm deep. Each monolith was subdivided into three depths (0–10, 10–20, and 20–30 cm). All visible roots in each soil layer were carefully collected by hand and stored in an ice box before transport to the laboratory for washing. Additional roots for analysis of Pi transporter genes were taken from the same plots for root morphology assessment. Roots of three plants per plot were carefully excavated and washed with water, separated from stems, and combined into one sample. The samples were preserved in liquid nitrogen for transport to laboratory. At the grain maturity stage (111 DAP), three plants per plot were harvested for determination of total shoot dry weight and P content. Grain yield was determined by manually harvesting and drying (at 60°C) ears from two rows per plot. At flowering and grain maturity stages, topsoil (0–20 cm) was sampled from each plot for analysis of soil Olsen-P and CaCl_2_-P.

### Sample analysis

We used Olsen-P as the indicator of plant available P in the calcareous soils used here [28] and CaCl_2_-P as the indicator of P loss risk [29]. Soil Olsen-P level was determined by the molybdo-vanadophosphate method based on extraction of air-dried soil with 0.5 M NaHCO_3_ (pH 8.5) at 25°C [28]. Soil CaCl_2_-P was measured by extracting air-dried soil with 0.01 M CaCl_2_ according to Hesketh and Brookes [29]. Plant P concentration was measured by the molybdo-vanadophosphate method after samples were digested with concentrated H_2_SO_4_ and H_2_O_2_ [30]. Plant P uptake was then calculated from plant dry weight and P concentration.

To measure root morphological traits, cleaned root samples were dispersed in water in a transparent array (30 cm×20 cm×2 cm) and imaged with a scanner (Epson Expression 1600, Seiko Epson, Nagano, Japan) at a resolution of 800 dpi. The images were analyzed by WinRhizo software (Regent Instrument Inc., Quebec, QC, Canada) to determine root length. Root dry weight was determined by weighing the oven-dried samples after scanning. Specific root length was calculated from root length and root dry weight, and root/shoot ratio was assessed from root dry weight and shoot dry weight, respectively.

AM colonization was measured in 1-cm fine root segments that had been thoroughly mixed. Roots were cleared with 10% KOH at 90°C for 1 h and stained with Trypan blue [31]. The percent root colonization by AM fungi was assessed by examining 30 randomly selected stained root segments at 100–400 × magnification with a light microscope according to Trouvelot *et al*. [32].

Six Pi transporters of the Pht1 family have been reported for maize: from *ZEAma;Pht1;1* to *ZEAma;Pht1;6* [33]. For simplification, these genes are presented as *ZmPht1;1* to *ZmPht1;6* here. Expression of these Pi transporter genes was analyzed in maize root samples by real-time quantitative RT-PCR (qRT-PCR) [34]. Total RNA was isolated from frozen root tissue using the Trizol reagent (cat. no. 15596018, Invitrogen, USA). The isolated RNA was treated with the RNase-Free DNase Set (cat. no. 79254, Qigen, Germany) to eliminate genomic DNA contamination before it was cleaned further with the RNeasy Plant Mini Kit (cat. no. 74904, Qigen, Germany). The first-strand cDNA was synthesized using the PrimeScript® RT reagent Kit Perfect Real Time (cat. no. DRR037A, Takara, Dalian) according to the manufacturer’s protocol. Then the qRT-PCR was performed on a Mastercycler Realplex4 Real Time PCR System (Eppendorf, Germany) based on the protocol of the SYBR® Premix EX TaqTM (cat. no. DRR041A, Takara) in 20 µl reaction volume, which contained 10 µl of SYBR Green PCR mix, 0.4 µM of each forward and reverse primer, 0.4 µg of diluted cDNA template, and the appropriate amounts of sterile double distilled water. The applied program was set as initial polymerase activation at 95°C, 30 s, then 40 cycles at 95°C, 5 s; 60°C, 35 s. The specificity of the PCR amplification was evaluated with a melt curve analysis from 60°C to 95°C following the final cycle of the PCR. All reactions were set up using four biological replicates. We used UBQ2 as the internal control gene as reported by Calderon-Vazquez *et al*. [34], and the transcription levels of each gene were normalized to that of UBQ2 by the 2^−△△Ct^ method. The sequences of the gene-specific primers used for the six transporters and UBQ2 were provided by L. Z. Long, pers. comm.:

*ZmPht1;1 primers*: - 5′-GACCCAGATGGTGTAGAATCGAACAT-3′, and

- 5′-TCACTTACTTTCCCGCCTATAACACACA-3′.

*ZmPht1;2 primers*: - 5′-GTCTGGTGAGGCTGAAGACTCAGAGG-3′, and

- 5′-ACATGATAGCCCACCATGTGCAGTGC-3′.

*ZmPht1;3 primers*: - 5′-TGTTTCCGTTCTGTCTGGTGCTTGTG-3′, and

- 5′-TCCCGACGGTGACCTCCGATTATTTA-3′.

*ZmPht1;4 primers*:*-* 5′-GAGACCCAGATGGTGTAGAGAATCG-3′, and

- 5′-CATCAAAACACAGCCAGGGTTGACT-3′.

*ZmPht1;5 primers*: - 5′-CCAAAGGTAAGTCGCTGGAAGAGAT-3′, and

- 5′-CCATTGCGTGCAACAAACAGTGAC-3′.

*ZmPht1;6 primers*: *-* 5′-CGGACGTGAGCAAGGATGACAA-3′, and

- 5′-GGATTCCACACCCCCTGTGTAGT-3′.

*ZmUBQ2 primers*: -5'- CTTTGCTGCTGCACGGGAGGAATG- 3', and

-5'- ATGGACGCACGCTGGCTGACTA-3'.

### Statistical analysis

Data are presented as means and standard errors (SE). One-way ANOVA (SPSS 13.0, USA) was conducted, and significant differences among means were determined by LSD at the *p* < 0.05 probability level. To explore the relationship between shoot dry weight and soil Olsen-P, we used relative shoot dry weight by normalizing shoot dry weight data according to the maximum value obtained at each sampling time for each experiment; the data were then fitted to a linear-plateau model using SAS statistical software (SAS 8.1, USA) [35]. The relationships between soil CaCl_2_-P, root morphological traits, AM colonization, expression of the six Pi transporter genes and soil Olsen-P were plotted using SigmaPlot statistical software (SigmaPlot 10.0, USA).

## Results

### Plant growth, P uptake, and risk of soil P loss

**Figure 1:**
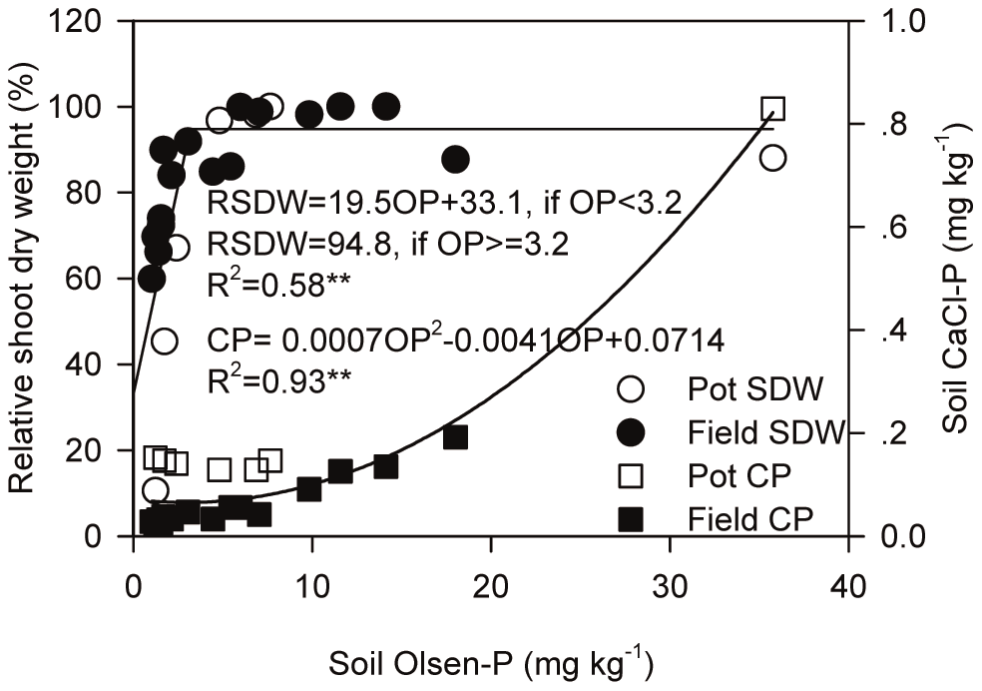
Maize growth and P loss risk in response to increasing soil P supply. Maize growth was presented as relative shoot dry weight, which was expressed relative to the highest mean value at each sampling time in each experiment. P loss risk was presented as soil CaCl_2_-P level. Abbreviations: RSDW: relative shoot dry weight; OP: Olsen-P; CP: CaCl_2_-P.

P application significantly increased soil P supply in terms of Olsen-P in both experiments, with the highest Olsen-P level in pot experiment being almost twice of that in the field (Table 1 and 2). Accordingly, shoot dry weight of 8-leaf plants in pot experiment increased fast with increasing P application up to 50 mg kg^−1^ soil, then increased only slightly and were reduced with further P supply (Table 1). Similar results were obtained for field plants at the flowering stage, with the critical P application rate for best shoot growth being 75 kg P ha^−1^ (Table 2). At maturity stage in the field, total shoot dry weight increased with increasing P supply, while grain yield plateaued when P application rate exceeded 75 kg P ha^−1^ (Table 2). Taken together, the result show that shoot dry weight was positively correlated with soil Olsen-P up to a threshold of 3.2 mg kg^−1^, at which value almost 95% of the relative shoot dry weight had been achieved (Figure 1).

**Table 1:** Agronomic traits of pot maize plants in response to different P application rates.

P rate	Olsen-P	CaCl_2_-P	Shoot dry weight	Shoot P concentration	P uptake
(mg P kg^−1^ soil)	(mg kg^−1^)	(mg kg^−1^)	(g plant^−1^)	(g kg^−1^)	(mg plant^−1^)
0	1.21 (0.11)^d^	0.15 (0.00)^b^	1.9 (0.1)^d^	1.06 (0.12)^e^	2.1 (0.2)^d^
12.5	1.71 (0.12)^d^	0.15 (0.00)^b^	8.3 (0.5)^c^	1.14 (0.12)^de^	9.4 (0.9)^cd^
25	2.39 (0.24)^d^	0.14 (0.01)^b^	12.4 (0.7)^b^	1.42 (0.04)^d^	17.6 (1.4)^c^
50	4.80 (0.88)^c^	0.13 (0.01)^b^	17.8 (1.3)^a^	1.94 (0.11)^c^	34.6 (2.5)^b^
75	6.86 (0.15)^b^	0.13 (0.01)^b^	18.1 (1.0)^a^	2.24 (0.05)^bc^	40.7 (3.0)^ab^
100	7.65 (0.87)^b^	0.15 (0.01)^b^	18.4 (2.2)^a^	2.43 (0.18)^b^	45.2 (8.3)^ab^
300	35.77 (0.88)^a^	0.83 (0.07)^b^	16.2 (0.7)^a^	3.18 (0.13)^a^	51.7 (4.3)^a^

Plants were sampled at the 8-leaf stage (56 days after planting).

Each value is the mean (± SE) of three replicates.

Values in a column followed by different letters are significantly different at *p* < 0.05.

**Table 2:** Agronomic traits of field maize plants in response to different phosphorus application.

DAP	P rate	Olsen-P	CaCl_2_-P	Shoot dry weight	Grain yield	Shoot P concentration	Grain P concentration	P uptake
	(kg P ha^−1^)	(mg kg^−1^)	(mg kg^−1^)	(Mg ha^−1^)	(Mg ha^−1^)	(g kg^−1^)	(g kg^−1^)	(kg ha^−1^)
57	0	1.02 (0.11)^e^	0.03 (0.00)^c^	4.01 (0.02)^c^		1.57 (0.14)^c^		6.29 (0.60)^d^
	12.5	1.39 (0.03)^e^	0.03 (0.00)^c^	4.43 (0.13)^c^		1.83 (0.21)^bc^		8.16 (1.05)^cd^
	25	1.67 (0.11)^e^	0.04 (0.00)^c^	6.02 (0.16)^b^		1.89 (0.11)^bc^		11.39 (0.69)^bc^
	50	3.04 (0.45)^de^	0.05 (0.00)^c^	6.15 (0.11)^b^		2.04 (0.15)^bc^		12.57 (1.09)^ab^
	75	5.97 (0.14)^cd^	0.06 (0.01)^c^	6.69 (0.29)^a^		2.12 (0.13)^b^		14.21 (1.20)^ab^
	100	7.03 (0.49)^c^	0.04 (0.00)^c^	6.62 (0.17)^a^		2.15 (0.30)^ab^		14.26 (2.14)^ab^
	150	11.57 (2.61)^b^	0.13 (0.04)^b^	6.70 (0.11)^a^		2.23 (0.23)^ab^		15.01 (1.77)^ab^
	300	18.00 (2.50)^a^	0.19 (0.03)^a^	5.87 (0.14)^b^		2.68 (0.13)^a^		15.75 (0.83)^a^
111	0	1.21 (0.01)^d^	0.02 (0.00)^c^	13.44 (0.68)^e^	2.97 (0.04)^d^	0.47 (0.02)^cd^	1.89 (0.05)^c^	10.57 (0.56)^e^
	12.5	1.53 (0.25)^d^	0.03 (0.00)^bc^	13.95 (1.19)^de^	5.06 (0.01)^c^	0.42 (0.02)^d^	1.95 (0.18)^c^	12.20 (0.52)^e^
	25	1.54 (0.25)^d^	0.02 (0.00)^c^	14.26 (0.50)^cde^	4.66 (0.04)^c^	0.41 (0.00)^d^	1.85 (0.01)^c^	12.60 (0.18)^e^
	50	2.12 (0.23)^d^	0.03 (0.01)^bc^	16.20 (0.20)^bcd^	6.08 (0.30)^b^	0.40 (0.02)^d^	2.25 (0.06)^b^	17.69 (0.61)^d^
	75	4.43 (0.17)^c^	0.03 (0.01)^bc^	16.36 (1.01)^bc^	7.12 (0.25)^a^	0.56 (0.03)^bc^	2.45 (0.11)^a^	22.73 (1.83)^c^
	100	5.41 (0.46)^c^	0.06 (0.01)^bc^	16.59 (0.95)^b^	7.41 (0.22)^a^	0.62 (0.06)^b^	2.41 (0.03)^ab^	23.54 (0.66)^bc^
	150	9.82 (0.67)^b^	0.09 (0.02)^ab^	18.93 (0.42)^a^	7.35 (0.58)^a^	0.62 (0.02)^b^	2.50 (0.07)^a^	25.48 (1.05)^b^
	300	14.12 (0.80)^a^	0.13 (0.06)^a^	19.29 (0.88)^a^	7.74 (0.20)^a^	0.75 (0.04)^a^	2.66 (0.07)^a^	29.07 (0.80)^a^

Each value is the mean (± SE) of four replicates.

Within each column and for each sampling time, values followed by different letters are significantly different at *p* < 0.05.

Abbreviation: DAP: days after planting.

In the pot experiment, shoot P concentration were similar until P application rate reached 50 mg kg^−1^ soil, above which it increased significantly with further P supply (Table 1). Contrarily, shoot P uptake in aboveground parts increased rapidly below P application rate of 50 mg kg^−1^ soil and then more slowly with higher P applications (Table 1). Similar responses were observed for field plants at both sampling stages, with a critical P application rate of 50 kg P ha^−1^ at flowering stage and 75 kg P ha^−1^ at maturity stage, respectively (Table 2).

The risk of soil P loss was indicated by the level of soil CaCl_2_-P. In the pot experiment, soil CaCl_2_-P level remained unchanged with P application rates between 0 and 100 mg P kg^−1^ soil and significantly increased only with the highest application rate (Table 1). Similarly, soil CaCl_2_-P in the field did not increase until P was applied at 150 kg P ha^−1^ at both sampling stages (Table 2). Overall, soil CaCl_2_-P level remained unchanged with soil Olsen-P level < 15 mg kg^−1^ (Figure 1).

### Responses of root morphology, AM colonization and expression of Pi transporter genes to soil P supply

**Figure 2:**
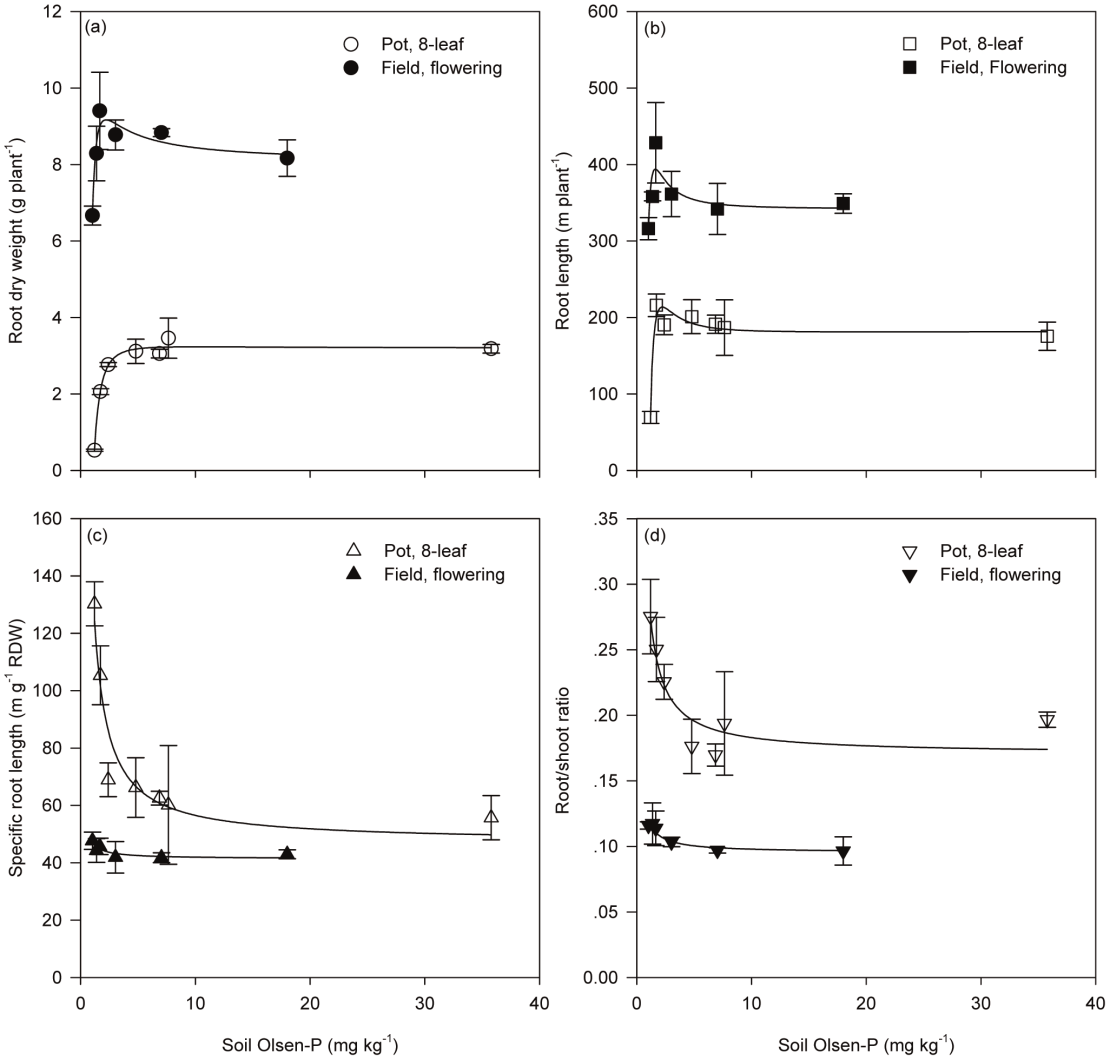
Root morphological traits in response to increasing soil P supply. In the pot experiment (open symbols), data were collected when plants were at the 8-leaf stage, and all visible roots in each pot were collected. In the field experiment (closed symbols), data were collected at the flowering stage, and roots were collected in a soil volume of 40 cm (row spacing) × 20 cm (plant spacing) × 30 cm (depth). Each symbol represents the mean (± SE) of three replicates for the pot experiment and four replicates for the field experiment, respectively. Abbreviation: RDW: root dry weight.

In the pot experiment, root dry weight increased as soil P supply increased, but plateaued once soil Olsen-P level reached 5 mg kg^−1^ (Figure 2a). In the field experiment, root dry weight also initially increased with increasing soil P supply, peaked when soil Olsen-P was about 2.5 mg kg^−1^, and then gradually declined to a plateau at an Olsen-P level around 10 mg kg^−1^ (Figure 2a). The responses of root length to P supply in both experiments were very similar to that of root dry weight of field plants, with the critical Olsen-P level indicating a plateau at 8 mg kg^−1^ (Figure 2b). As soil Olsen-P increased from very low levels, specific root length and root/shoot ratio of pot plants declined substantially at first, and then gradually reached a plateau when Olsen-P exceeded 8 mg kg^−1^; field plants showed similar responses, but decreases were not so pronounced compared with those of pot plants, and both traits reached the plateau at a lower critical Olsen-P level about 5 mg kg^−1^ (Figure 2c, d). Generally, specific root length and root/shoot ratio were much higher for pot-grown plants than field-grown plants.

**Figure 3:**
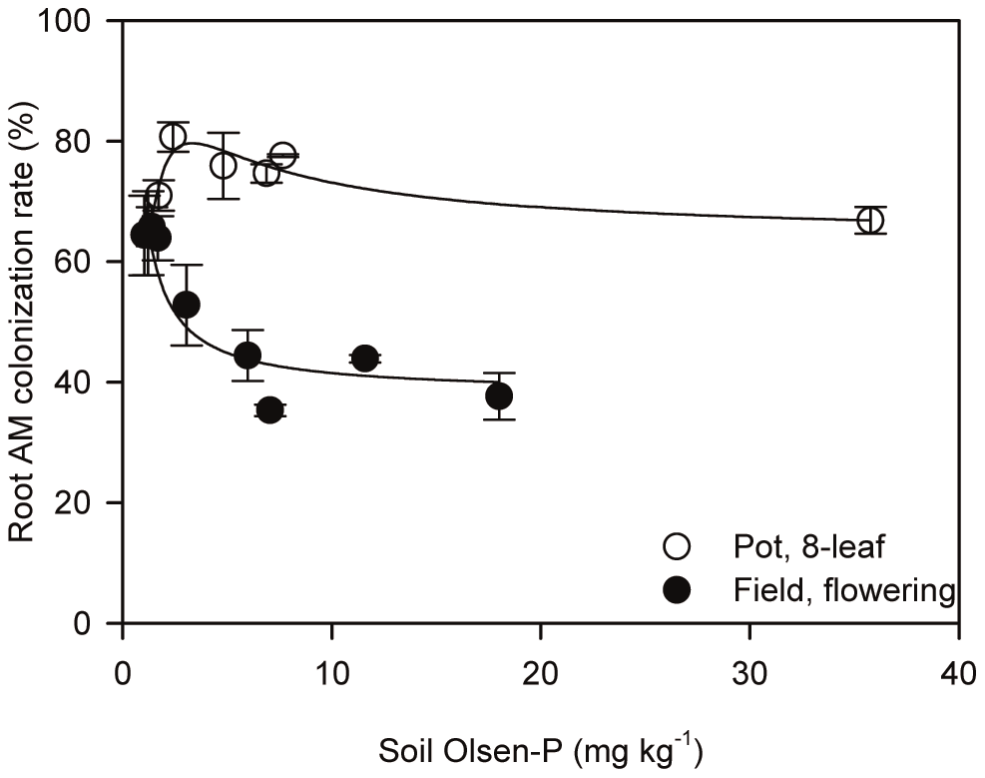
Root AM colonization in response to increasing soil P supply. In the pot experiment (open symbols), data were collected when plants were at the 8-leaf stage. In the field experiment (closed symbols), data were collected at the flowering stage. Each symbol represents the mean (± SE) of three replicates for the pot experiment and four replicates for the field experiment, respectively. Abbreviation: AM: arbuscular mycorrhizal.

In the pot experiment, root AM colonization of plants at the 8-leaf stage initially increased with increasing soil P supply, peaked at an Olsen-P level about 2.5 mg kg^−1^, and then tended to gradually decrease until Olsen-P level reached 10 mg kg^−1^, above which AM colonization remained stable at an average value of 70% with further P supply (Figure 3). In the field at the flowering stage, with an increase of soil Olsen-P root AM colonization declined rapidly at first, and gradually plateaued at 40% when Olsen-P level exceeded 5 mg kg^−1^ (Figure 3). AM colonization rates were generally higher for pot plants than field plants.

**Figure 4:**
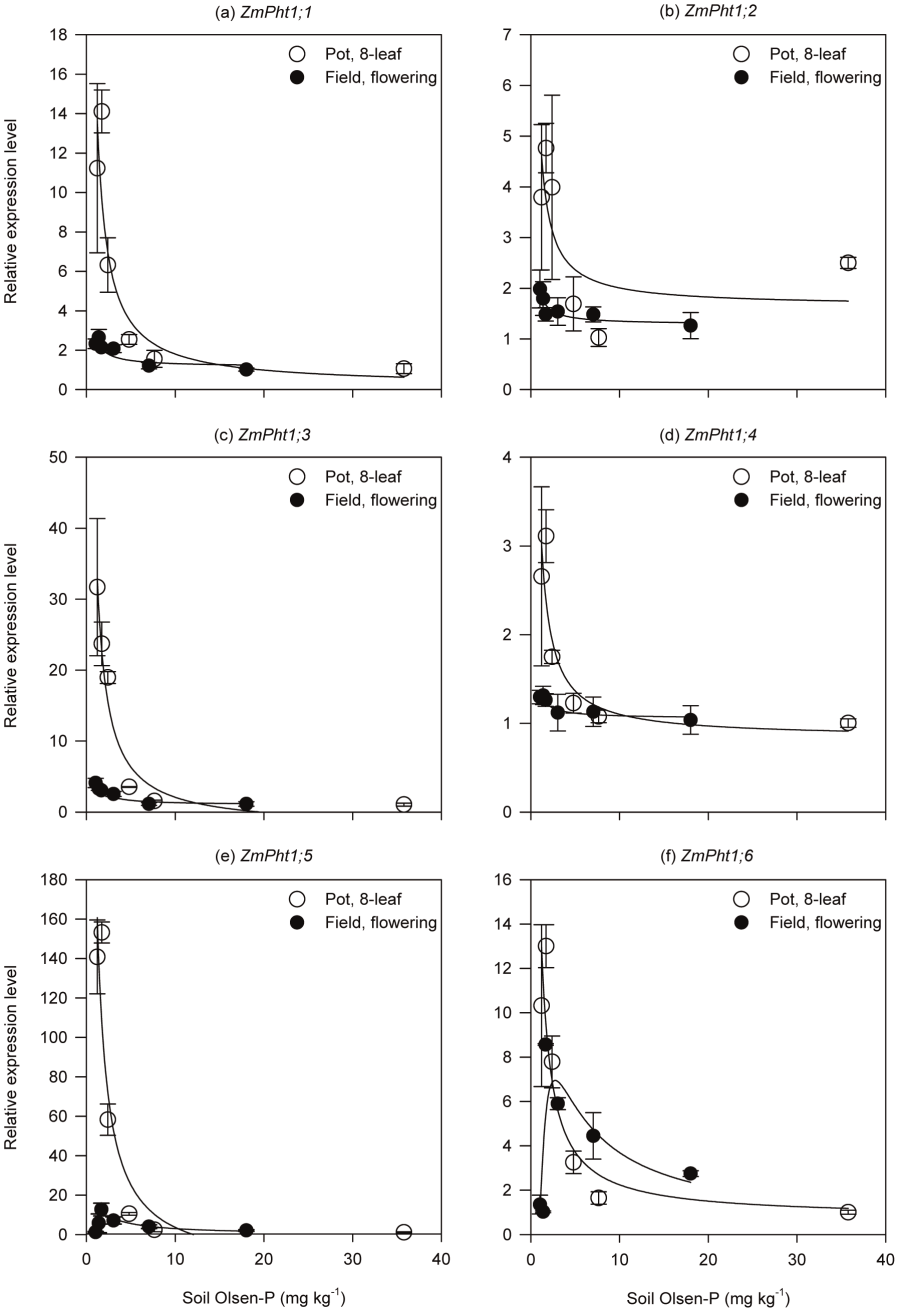
Expression of root Pht1 transporter genes in response to increasing soil P supply. Roots were sampled at the 8-leaf stage for pot plants (open symbols) and at the flowering stage for field plants (closed symbols). Gene relative expression level was measured by real-time quantitative RT-PCR. UBQ2 was used as the internal control. For each gene, the lowest expression level was set equal to 1.0. Each symbol represents the mean (± SE) of three replicates for the pot experiment and four replicates for the field experiment, respectively.

We detected expressions of all the six Pht1 Pi transporter genes in both experiments. For pot plants, the six genes responded similarly to soil P supply, i.e. their transcript levels initially decreased rapidly with increasing P supply until soil Olsen-P level reached about 10 mg kg^−1^, above which their expression kept very low and stable (Figure 4). Results were more complex in the field experiment. Expressions of *ZmPht1;1* to *ZmPht1;4* had similar responses to increase of soil Olsen-P, which were in accord with their expressions in pot plants, with the critical Olsen-P level indicating up-regulation approximating 5 mg kg^−1^ (Figure 4a–c). The transcript level of *ZmPht1;5* increased with increasing soil Olsen-P, peaked around 2.5 mg kg^−1^, and then gradually declined to a stable status at Olsen-P level about 8 mg kg^−1^ (Figure 4e). Expression response of *ZmPht1;6* was similar to that of *ZmPht1;5* when soil Olsen-P was below 8 mg kg^−1^, but declined further above this level. Generally, the expression responses of the six genes to soil P supply were more significant in the pot experiment than those in the field experiment.

## Discussion

Many studies have reported that as soil P supply increased from initially low levels, crop yield increases quickly at first and then more slowly to an asymptote [36], [37], [38]. Based on this typical response, it has been proposed that there is a critical level of soil P for optimum crop yield [38], [39]. Under the conditions of our study, the critical level of soil P for maize production was 3.2 mg kg^−1^ (Olsen-P) (Figure 1). This value is relatively low as the critical P values reported in other studies have ranged from 3.9 to 17.3 mg kg^−1^ for maize production [35], [39], [40], [41].

To maximize crop yield, farmers tend to increase soil P and maintain it at a level that is greater than the critical value required for optimum yield [24]. Soil, however, cannot retain unlimited quantities of P. When soil P rise to a certain point, the environmental risk threshold, the risk of P loss increases significantly; and P loss may cause water pollution [7], [42]. As a result, P management has developed to embraces both agronomic and environmental goals, with the environmental risk threshold used as the building-up threshold for soil P [43], [44], [45]. Using CaCl_2_-P as an indicator of P loss risk, we found that the environmental risk threshold in terms of Olsen-P, which resulted in a significant increase in CaCl_2_-P, was about 15 mg kg^−1^ in this study (Figure 1). This value is in the range of 10–119 mg kg^−1^ previously reported for a range of soils [29]. Therefore, based on the current P management goals of maximizing crop yield and minimizing adverse environmental effects, our results indicate that soil Olsen-P level should be maintained between 3.2 and 15 mg kg^−1^ for maize production under our experimental conditions.

When soil Olsen-P decreased from 10 to 3.2 mg kg^−1^, shoot growth was maintained but root length (and probably root dry weight) gradually increased (Figure 2a, b), so did root surface area (data not shown), whilst specific root length and root/shoot ratio remained relatively low and stable. This suggests that maize root growth was more sensitive than shoot growth to the reduction in soil P supply, and that maize root morphology acclimated to a reduced P supply firstly by increasing root length (and probably root dry weight and root surface area). When soil P supply was further reduced below 3.2 mg kg^−1^, increases in specific root length and in the root/shoot ratio appeared to be the main morphological adaptation (Figure 2c, d). These results are consistent with commonly observed responses of plants to P limitation: plants allocate more biomass to root and produce more root length per unit of metabolic investment in root tissue in order to improve the capacity of roots to explore soil [22], [46]. Soil Olsen-P level higher than 10 mg kg^−1^ showed an inhibitory effect on all the morphological traits we measured.

For AM plants like maize, there are two P uptake pathways. One is the direct P uptake via root epidermis and root hairs. Among the six Pi transporters in Pht1 family reported for maize, *ZmPht1;1*–*ZmPht1;4* are considered to facilitate the direct uptake pathway [33], although the affinity for Pi of these four transporters remains uncertain. Phylogenetic analysis shows that *ZmPht1;1*, *ZmPht1;2*, and *ZmPht1;4* are closely related with rice *OsPT8* while *ZmPht1;3* closely clusters with rice *OsPT6* [33]. Both *OsPT6* and *OsPT8* are identified high-affinity Pi transporters [47]. Thus we speculate that *ZmPht1;1* to *ZmPht1;4* may also play the role of high-affinity Pi transporters. In the current study we found that these four genes responded similarly to soil P supply, and the up-regulation of their expressions was induced at an Olsen-P level (10 and 5 mg kg^−1^ in pot and field experiments, respectively) higher than the critical level for shoot growth (Figure 1, 4a–d), suggesting that root response on the molecular level to reduced P supply occurred at a higher P level than shoot growth. The up-regulation of the expressions for the four genes coincided with the increase of root length until soil Olsen-P was reduced to 2.5 mg kg^−1^, from which root length began to decrease with further P supply reduction (Figure 2b, 4a–d). Such responses probably indicate the cooperation between root morphology and physiology for efficient P acquisition in the direct pathway when soil P supply reduced from sufficiency to deficiency. We noticed the overall up-regulation levels of these four genes were significantly higher in pot-grown plants than field-grown plants. One reason may be that the pot plants suffered relatively more P deficiency at lower P supply as indicated by the lower shoot P concentrations of pot plants than those of field plants (Table 1 and 2).

The other P uptake pathway is through AM symbioses. The rate of root colonization by AM fungi is considered an important factor for the extent to which AM symbioses contribute to P uptake and growth response of AM plants [48]. The soil P level plays an important role in influencing AM colonization [49], [50], [51]. In the current study, we found the influence of soil P on AM colonization was strongest when soil Olsen-P level was below 10 mg kg^−1^ (Figure 3), indicating a threshold of soil P supply for AM colonization as reported by others [51], [52]. As Olsen-P reduced from 10 to 3.2 mg kg^−1^, the increasing root AM colonization suggests that the mycorrhizal pathway was enhanced to increase P acquisition without loss of shoot growth.

There are two types of Pht1 transporter genes whose expression can be induced in mycorrhizal roots: AM-specific Pi transporter genes which are expressed strictly in response to AM symbioses, and AM-inducible Pi transporter genes which can be strongly induced by AM symbioses but have a basal expression in non-mycorrhizal roots [53]. *ZmPht1;6* has been identified as an AM-inducible Pi transporter gene for maize [33]. Currently there is no report regarding an association between *ZmPht1;5* and AM symbioses. *ZmPht1;5*, however, clusters with *OsPT13* on the phylogenetic tree [33], [53], and *OsPT13* is an AM-inducible gene in rice [54], possibly indicating a role of *ZmPht1;5* in the mycorrhizal pathway. Coinciding with the response of root AM colonization, the expression levels of the two genes were gradually up-regulated when soil Olsen-P was reduced from 10 to 3.2 mg kg^−1^ in both experiments (Figure 4e, f). When soil P supply further decreased below the critical level for shoot growth, we noticed that both AM colonization and expressions of the two genes in the pot experiment were different from those in the field study. This may be partly in relation to the dependency on mycorrhizal pathway for P uptake under P deficiency when maize plants were at different growth stages. In the early growth season, root growth and development is relatively poor, but plant requirement of P is high to support fast growth [55], [56]. Thus an effective AM association may help to improve P nutrition for young plants. Our data showed that pot plants at the 8-leaf stage had much less root length (Figure 2b) and root surface area (data not shown) for P acquisition compared with field plants at the flowering stage. To compensate the direct pathway, the younger plants in the pot experiment might depend more on mycorrhizal pathway through enhancing expression of AM-inducible Pi transporter genes but limiting fungal growth as represented by reduced AM colonization, possibly a strategy to save carbon for efficient P acquisition. Besides, the much smaller soil volume for root growth and exploration in the pot experiment compared with field conditions may trigger pot plants to depend more on the mycorrhizal pathway under P deficiency, as indicated by the overall higher AM colonization rates and up-regulation levels of AM-inducible Pi transporter genes.

In summary, under the conditions of our study, most root adaptations (root morphology, AM colonization and expression of Pi transporter genes) involved with P acquisition were triggered at Olsen-P level < 10 mg kg^−1^. Therefore, to maximize the inherent potential of maize roots to obtain P, we suggest that the upper level of optimum soil P supply in present study should be reduced to 10 mg kg^−1^, rather than the environmental risk threshold of 15 mg kg^−1^.

Soil P status varies across the world’s croplands, and P deficits occur on 30–40% of the world’s arable land [57], especially in Africa and South Asia. Sustainable crop production on these soils requires some level of P fertilization, a level that can optimize agronomic goals while maximizing inherent potential of roots to obtain P efficiently. In contrast to areas with P-depleted soils, most areas with intensive agriculture (Western Europe, North America, and East Asia) have the opposite problem in that levels of soil P have reached or exceeded the levels required by crops. In these soils, management should prevent further increases in P level or reduce P level not only to reduce environmental risk but also to better use the inherent potential of roots to acquire P. Although the importance of developing cultivars with an increased ability to obtain and utilize P is now recognized, our study illustrates that improvement of agronomic soil P management will also be important to explore the inherent potential of roots for efficient P acquisition.
